# Peer Review of “Use of Smartphone Apps for Improving Physical Function Capacity in Cardiac Patient Rehabilitation: Systematic Review”

**DOI:** 10.2196/33180

**Published:** 2021-09-17

**Authors:** Alexis Beatty

**Affiliations:** 1 School of Medicine University of California, San Francisco San Francisco, CA United States

**Keywords:** cardiac rehabilitation, physical capacity, exercise, smartphone apps

*This is a peer-review report submitted for the paper “Use of Smartphone Apps for Improving Physical Function Capacity in Cardiac Patient Rehabilitation: Systematic Review”.*

## Review Round 1

### General Comments

This paper [1] reviews published studies on use of smartphone apps for cardiac rehabilitation (CR). The authors reach interesting conclusions about integrating devices for monitoring physical activity and vital signs, which would be the main contribution of this paper. However, there are significant issues with the methods and a number of incorrect statements within the manuscript that are of concern.

### Specific Comments

#### Major Comments

1. Please justify your statement in the abstract that apps reduce the cost of CR, or if unable to justify or cite a reference, remove this statement.

2. Would suggest citing newer references and original references for outcomes and rates of participation in CR: Ritchey MD, Maresh S, McNeely J, Shaffer T, Jackson SL, Keteyian SJ, Brawner CA, Whooley MA, Chang T, Stolp H, Schieb L, Wright J. Tracking cardiac rehabilitation participation and completion among Medicare beneficiaries to inform the efforts of a national initiative. Circ Cardiovasc Qual Outcomes 2020;13(1):e005902.

3. Would suggest reading and referencing the Home-Based Cardiac Rehabilitation Scientific Statement: Thomas RJ, Beatty AL, Beckie TM, Brewer LC, Brown TM, Forman DE, Franklin BA, Keteyian SJ, Kitzman DW, Regensteiner JG, Sanderson BK, Whooley MA. Home-based cardiac rehabilitation: a scientific statement from the American Association of Cardiovascular and Pulmonary Rehabilitation, the American Heart Association, and the American College of Cardiology. Circ 2019;140(1):e69-e89.

4. Your search strategy appears rather limited. Would suggest including additional terms, such as *mobile app*, *mobile phone*, and *digital health*.

5. You should exclude studies that were not randomized per your methods (eg, Forman [2], Layton [3], Worringham [4]).

6. You should distinguish between lack of improvement with smartphone CR and lack of significant difference versus CR—they are different things.

7. Your definition of CR phases is not how most view it—Phase I is typically thought of as inpatient, and it is now recommended that patients start Phase 2 within 21 days and participate for 12 weeks.

8. I suggest including “Phase of rehab” in your table rather than in the text.

9. In your table, you should display the numbers of the outcome measure rather than the word description of the comparison.

## Review Round 2

The authors appropriately responded to comments and the revised manuscript is significantly improved.

## Abbreviations

CR: cardiac rehabilitation
